# Prevalence of epiretinal membrane in the phakic eyes based on spectral-domain optical coherence tomography

**DOI:** 10.1371/journal.pone.0245063

**Published:** 2021-01-07

**Authors:** Boyun Kim, Ayoung Choi, Jin Heung Park, Sohee Jeon

**Affiliations:** Keye Eye Center, Seoul, Korea; Massachusetts Eye & Ear Infirmary, Harvard Medical School, UNITED STATES

## Abstract

The prevalence of epiretinal membrane (ERM) and associated factors in the phakic eyes have not been fully elucidated yet. This cross-sectional study included 2,354 phakic eyes without retinal diseases or surgical history. Ocular parameters, such as uncorrected distance visual acuity (UDVA), corrected distance visual acuity (CDVA), spherical equivalent (SE), intraocular pressure (IOP), white-to-white corneal diameter (WTW), mean keratometric value (Km) of total corneal refractive power at 4-mm diameter (TCRP4), astigmatism of TCRP4, total corneal irregular astigmatism (TCIA), pupil diameter, axial length (AXL), anterior chamber depth (ACD), lens thickness (LT), and posterior vitreous detachment (PVD) were compared between ERM group and control group. Additionally, an age-matched control group was selected by individual matching and compared with the ERM group to eliminate the confounders. Multiple logistic regression analysis was performed to evaluate the factors associated with the presence of ERM. Among 2,354 eyes, 429 eyes (18.2%) had ERM based on spectral-domain optical coherence tomography. The ERM group showed higher prevalence of PVD, worse CDVA, higher astigmatism of TCRP4, higher TCIA, smaller pupil size, longer AXL, and thicker LT than control group (P < 0.001, P < 0.001, P = 0.011, P < 0.001, P = 0.023, P < 0.001, and P < 0.001, respectively). Only PVD, CDVA, SE, astigmatism of TCRP4, TCIA, and AXL maintained the significance when compared with the age-matched control group (P < 0.001, P = 0.026, P < 0.001, P = 0.001, P = 0.003, and P < 0.001, respectively). Multivariate logistic regression analysis showed that age, PVD, CDVA, and TCIA were independently associated with the presence of ERM (P < 0.001, P < 0.001, P = 0.011, and P = 0.002). The prevalence of ERM detected using SD-OCT was 18.2% in the middle aged phakic population. Eyes with TCIA, in addition to older age and PVD, were more likely to have ERM.

## Introduction

Epiretinal membrane (ERM) is a condition of cellular proliferation at the inner retinal surface [1, 2], which has various effects on the visual quality based on its location, thickness, transparency, and contractility [3, 4]. The prevalence of ERM reportedly ranged from 1.02% in Chinese population [5] to 28.9% in multiethnic U.S. population [6] based on fundus photography, however, the prevalence significantly increased up to 69.9% in French population because the resolution of macular images markedly improved due to the introduction of spectral-domain optical coherence tomography (SD-OCT) [7, 8].

Although ERM is a highly prevalent disease [5, 6], the pathophysiology of ERM, especially primary ERM, is not yet fully understood. Known demographic risk factors for the development of primary ERM are age and cataract surgery, which were consistently shown in many large-scale studies [7–12]. In a recent longitudinal study using SD-OCT, the development of ERM based on aging was associated with the vitreoretinal interface (VRI), such as vitreomacular or vitreopapillary adhesion at baseline [13]. In addition, the anteroposterior movement of vitreous during cataract surgery has been hypothesized as the main cause of ERM progression in eyes that underwent cataract surgery, indicating posterior vitreous detachment (PVD) an important factor for the progression of ERM [14].

In a previous study in which the PVD in healthy human eyes was evaluated using OCT, an incomplete or partial PVD began as early as the fourth decade of life and slowly progressed to an advanced stage in subsequent decades [15]. In most studies, ERM [5–8] and PVD [16–18] were evaluated in an older cohort, around the seventh decade of life. Only limited information exists regarding ERM and PVD in middle-aged subjects in the fifth and sixth decade of life, which is presumably the period when ERM [5, 6] and PVD start [15]. Therefore, in the present study, the prevalence of ERM and the correlation with various ocular parameters in phakic eyes of otherwise healthy patients mainly in their fifth and sixth decade of life were evaluated.

## Materials and methods

### Participants

A cross-sectional study was performed on phakic subjects who underwent SD-OCT for presbyopic correction or cataract surgery at the KEYE Eye Center, Seoul, Korea between February 2018 and July 2020. Patients with certain retinal diseases other than ERM, such as age-related macular degeneration, diabetic retinopathy, retinal vascular occlusions, or a previous history of any ocular surgery including cataract, refractive or vitreoretinal surgery were excluded. During the 29 months, according to our database, 4,174 patients (8,348 eyes) visited our clinic for decreased distant and/or near vision. Among those 8,348 eyes, 5,474 eyes with surgical history were not screened for full ophthalmologic evaluation, leaving 2,874 eyes with full ophthalmologic evaluation. Among those 2,874 eyes with data, 35 eyes were excluded from low image quality from corneal or lens opacity. Total 485 eyes with significant retinal diseases, such as age-related macular degeneration, diabetic retinopathy, retinal vascular occlusion, or retinal detachment were excluded for further analysis. The study was approved by the Institutional Review Board (IRB)/Ethics Committee of KEYE EYE Center (IRB number 20200724–001). The study protocol adhered to the tenets of the Declaration of Helsinki.

### Ocular examination

The ocular screening included monocular uncorrected distance visual acuity (UDVA), corrected distance visual acuity (CDVA), refraction, intraocular pressure (IOP) measurement, biomicroscopic examination, indirect binocular ophthalmoscopy, optical biometry with a partial coherence interferometry device (IOL Master, Carl Zeiss Meditec, Jena, Germany), corneal topography using the Pentacam Scheimpflug System (Oculus Inc., Berlin, Germany), ultra-widefield fundus photography (Optos Optomap Panoramic 200A Imaging System, Optos plc, Dunfermline, Scotland), SD-OCT version 5 (Heidelberg Engineering, Heidelberg, Germany), and Cirrus high definition-optical coherence tomography (HD-OCT; Carl Zeiss Meditec, Inc., Dublin, CA, USA).

#### Definitions

The ERM severity was graded as previously defined by Delyfer et al. [8]: (1) stage 1 or continuous hyperreflectivity, presence of a continuous hyperreflective signal at the inner retinal surface on at least three consecutive sections of the macular cube; (2) stage 2 or mature ERM without foveal involvement, stage 1 associated with retinal folds but without alterations of the foveal depression; (3) stage 3 or mature ERM with foveal involvement, stage 2 associated with foveal depression alterations.

The vitreomacular interface (VMI) was classified as previously defined by the International Vitreomacular Traction Study (IVTS) Group [19]: (1) no PVD; (2) focal vitreomacular adhesion (VMA) ≤ 1,500 μm; (3) broad VMA > 1,500 μm; (4) focal vitreomacular traction (VMT) ≤ 1,500 μm; (5) broad VMT > 1,500 μm; (6) PVD. VMA was defined as an elevation of the cortical vitreous above the retinal surface, with the vitreous remaining attached within a 3-mm radius of the fovea. VMT was defined when there was an association of attachment with the distortion of the foveal structure. For binary analysis, eyes were divided into the no PVD group (type 1) and detachment group (type 2–6). Two investigators (JHP and BK) who were masked to the patient groups and identities graded the ERM severity and PVD classification.

#### Statistical analysis

Eyes with ERM were classified as ‘ERM group’, while eyes without ERM were classified as ‘Control group’. To eliminate confounders, we randomly selected an ‘Age-matched control group’ by ‘individual matching’. Student’s t-test or Mann-Whitney U test was used for comparison of continuous variables between groups. Chi-square test was used for the comparison of categorical variables between groups. Multiple logistic regression analysis was performed to evaluate the association of ocular characteristics with ERM. Visual acuity was recorded as a Snellen value and then converted to logarithm of the minimum angle of resolution (logMAR) scale for statistical analysis. Uncorrected distance visual acuity (UDVA), corrected distance visual acuity (CDVA), spherical equivalent (SE), intraocular pressure (IOP), white-to-white diameter (WTW), mean keratometric value (Km) of total corneal refractive power at 4-mm diameter (TCRP4), astigmatism of TCRP4, total corneal irregular astigmatism (TCIA), pupil diameter from Pentacam Scheimpflug System, axial length (AXL), anterior chamber depth (ACD), and lens thickness (LT) using optical biometry with a partial coherence interferometry device were evaluated.

SPSS, version 15.0 for Windows (SPSS, Inc., Chicago, IL, USA) was used for statistical analysis. Descriptive data were recorded as means ± standard deviation (SD) unless otherwise specified. A P-value < 0.05 was considered statistically significant.

## Results

Among the 2,874 eyes from 1,437 subjects with SD-OCT images during the study period, 520 eyes were excluded due to retinal diseases or media opacity that limited retinal examination, resulting in 2,354 eyes for further analysis. Among the 2,354 eyes, 429 eyes (18.2%) from 297 patients showed ERM and 1,925 eyes (81.8%) showed no ERM based on SD-OCT (Table 1 and S1 Table). Among the 297 patients, 136 patients had bilateral ERM.

**Table 1:** Comparison of clinical characteristics of ERM and control group.

Characteristics	All population (n = 2354)	ERM group (n = 429)	Control group (n = 1925)		Age-matched control group (n = 429)	
	Data	Data	Data	P value	Data	P value
Age, years	58.72 ± 5.73	62.24 ± 5.31	57.94 ± 5.52	<0.001*	62.15 ± 5.19	0.800*
Gender, male (%)	1180 (50.1)	211 (49.2)	969 (50.3)	0.670^†^	228 (53.1)	0.304^†^
PVD, yes (%)	1617 (68.7)	420 (97.9)	1197 (62.2)	<0.001*	320 (74.6)	<0.001*
UDVA, LogMAR	0.34 ± 0.30	0.35 ± 0.27	0.33 ± 0.31	0.327*	0.35 ± 0.29	0.689*
CDVA, LogMAR	0.03 ± 0.10	0.06 ± 0.11	0.03 ± 0.09	<0.001*	0.04 ± 0.12	0.026*
SE, D	-0.15 ± 2.74	-0.32 ± 3.29	-0.11 ± 2.61	0.215*	0.51 ± 2.62	<0.001*
IOP, mmHg	15.43 ± 2.68	15.83 ± 2.87	15.75 ± 2.79	0.816*	15.87 ± 2.80	0.904*
WTW, mm	11.40 ± 0.38	11.37 ± 0.37	11.41 ± 0.38	0.132*	11.38 ± 0.39	0.661*
Km of TCRP4, D	43.64 ± 1.53	43.57 ± 1.73	43.65 ± 1.49	0.393*	43.69 ± 1.38	0.235*
Astigmatism of TCRP4, D	0.79 ± 0.56	0.86 ± 0.65	0.77 ± 0.54	0.011*	0.73 ± 0.48	0.001*
TCIA, D	0.16 ± 0.07	0.18 ± 0.09	0.15 ± 0.06	<0.001*	0.16 ± 0.07	0.003*
Pupil diameter, mm	3.69 ± 0.72	3.61 ± 0.77	3.70 ± 0.71	0.023*	3.65 ± 0.70	0.081*
AXL, mm	23.82 ± 1.27	24.03 ± 1.58	23.78 ± 1.19	<0.001*	23.68 ± 1.11	<0.001*
ACD, mm	3.16 ± 0.35	3.17 ± 1.09	3.17 ± 0.34	0.993*	3.13 ± 0.32	0.426*
LT, mm	4.44 ± 0.32	4.52 ± 0.31	4.42 ± 0.32	<0.001*	4.50 ± 0.30	0.406*

ACD = anterior chamber depth; AXL = axial length; CDVA = corrected distance visual acuity; IOP = intraocular pressure; ERM = epiretinal membrane; Km = mean keratometric value; LT = lens thickness; PVD = posterior vitreous detachment; SE = spherical equivalent; TCIA = total corneal irregular astigmatism; TCRP4 = total corneal refractive power at 4 mm; UDVA = uncorrected distance visual acuity; WTW = white to white corneal diameter. Data are mean ± standard deviation unless otherwise indicated.

*Student t test or Mann-Whitney U test.

^†^x^2^ or Fisher exact test.

**Fig 1:**
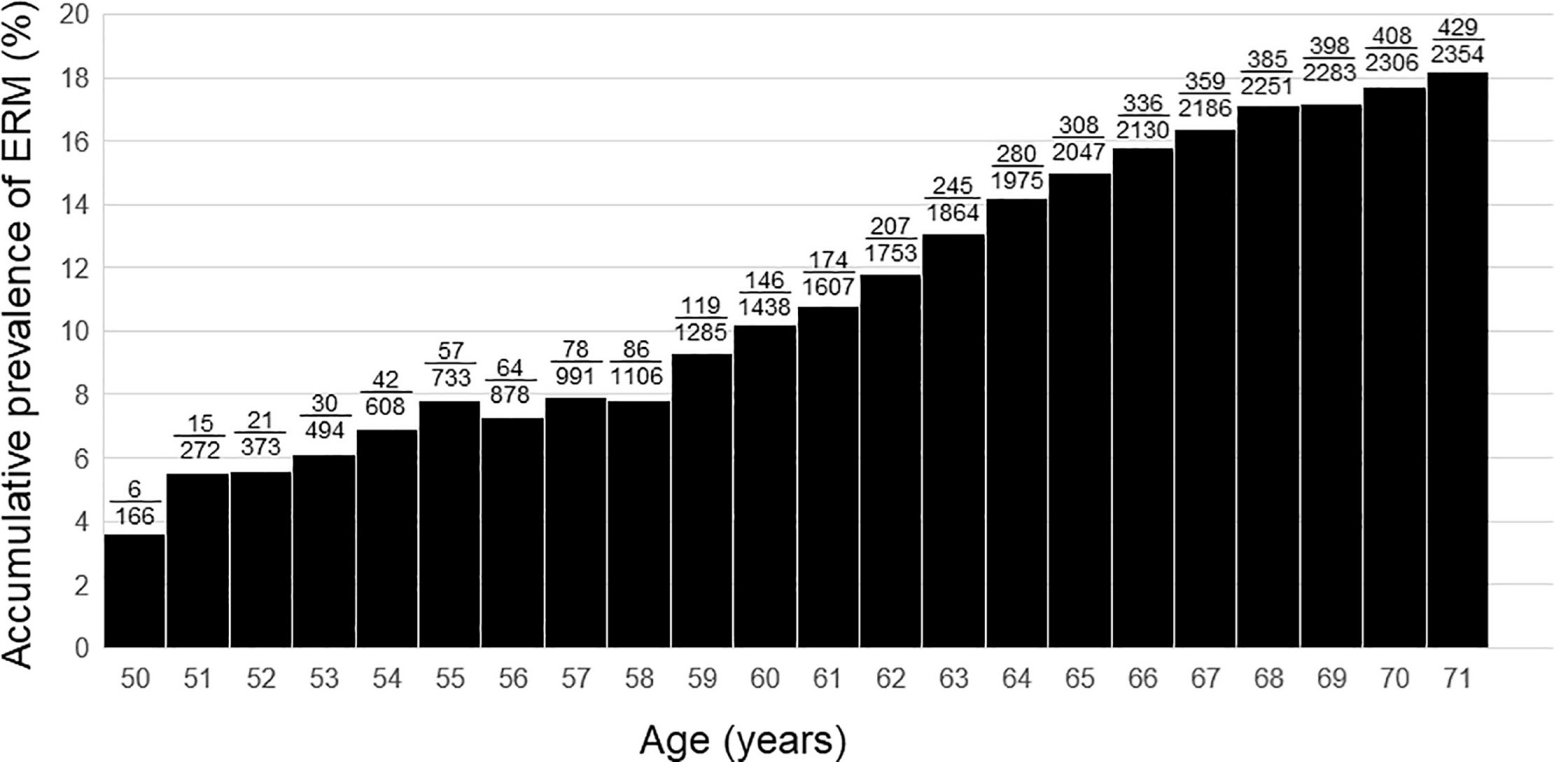
Bar graphs showing the accumulative prevalence of epiretinal membrane (ERM) based on age. The fractional numbers above the bar indicate the number of eyes with ERM/total number of eyes.

The mean age of the study population was 58.72 ± 5.73 years. The ERM group was older than control group (62.24 ± 5.31 years and 57.94 ± 5.52 years, respectively; P < 0.001). The prevalence of ERM continuously increased from 3.6% to 18.2% with age in phakic eyes, showing a relationship between age and prevalence of ERM (Fig 1). The ERM group showed higher prevalence of PVD, worse CDVA, higher astigmatism of TCRP4, higher TCIA, smaller pupil size, longer AXL, and greater lens thickness as compared to control group (P < 0.001, P < 0.001, P = 0.001, P < 0.001, P = 0.023, P < 0.001, and P < 0.001, respectively; Table 1). Difference in gender, UDVA, SE, IOP, WTW, Km of TCRP4, and ACD was not observed (P = 0.670, P = 0.327, P = 0.215, P = 0.816, P = 0.132, P = 0.393, and P = 0.993, respectively).

Because age can affect various ocular parameters, such as increasing against-the-rule astigmatism with age, age-matched controls were selected from enrolled subjects for comparison of factors other than age in this phakic population (age-matched control group; Table 1). Difference in age and gender was not observed between the groups (P = 0.800 and P = 0.304, respectively). The pupil diameter and LT were not statistically significant (P = 0.081 and P = 0.406), however, SE showed statistical significance when compared with age-matched control group (P = 0.215 and P < 0.001, respectively). The prevalence of PVD, CDVA, astigmatism of TCRP4, TCIA, and AXL still showed significant difference when compared with the age-matched control group (P < 0.001, P = 0.026, P = 0.001, P = 0.003, and P < 0.001, respectively).

**Fig 2:**
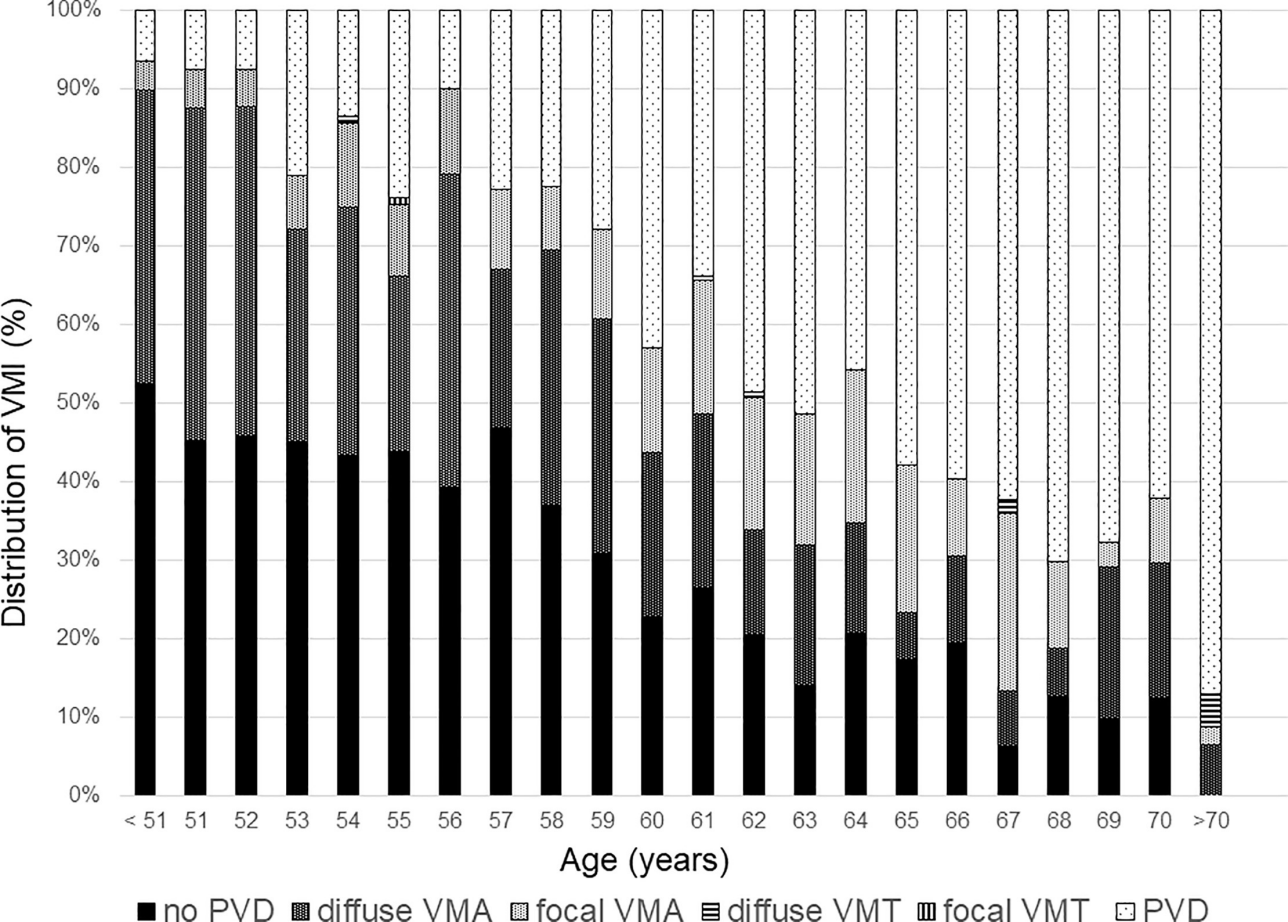
Bar graphs showing the distribution of vitreomacular interface based on age. VMI = vitreomacular interface; PVD = posterior vitreous detachment; VMA = vitreomacular attachment; VMT = vitreomacular traction.

**Fig 3:**
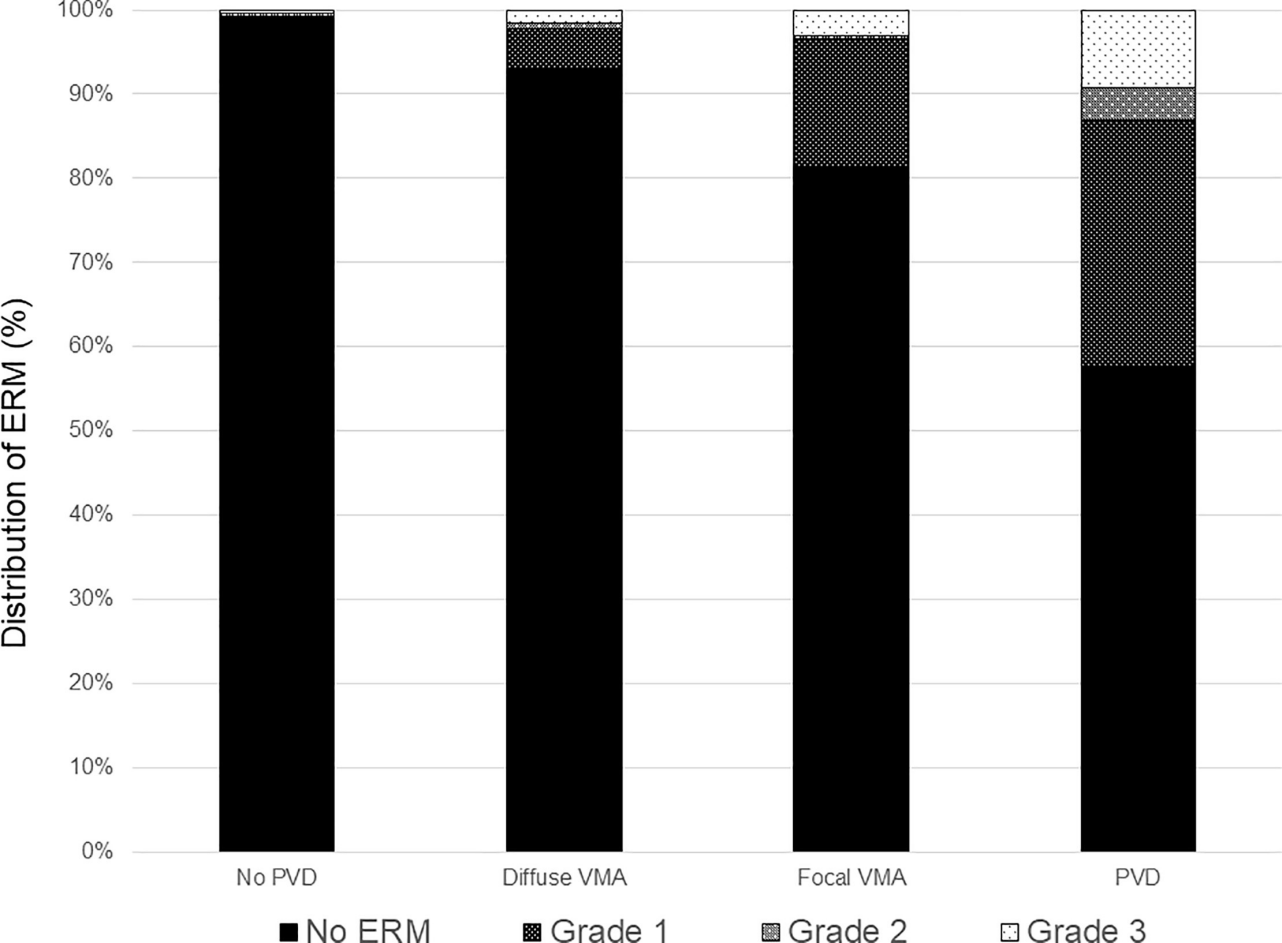
Bar graphs showing the distribution of epiretinal membrane based on vitreomacular interface status. ERM = epiretinal membrane; PVD = posterior vitreous detachment; VMA = vitreomacular attachment.

**Fig 4:**
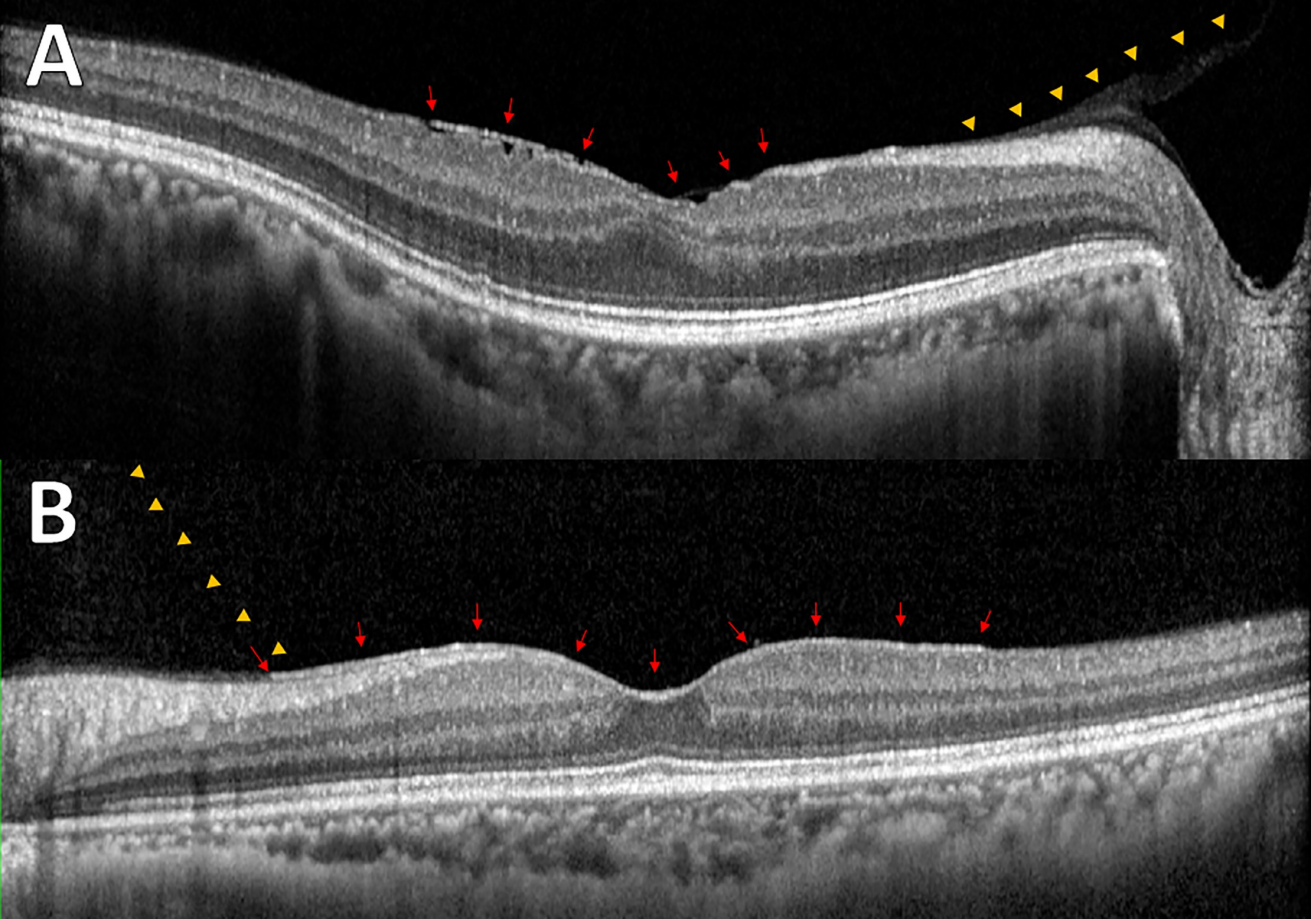
Representative images from enrolled subjects with epiretinal membrane without posterior vitreous detachment. (A) Membranous changes (arrows) were shown with the posterior hyaloid membrane (PHM; arrowheads) and wrinkling of underlying retina. (B) Even membranous changes (arrows) were observed between PHM (arrowheads) and inner retina without structural changes in the underlying retina.

**Fig 5:**
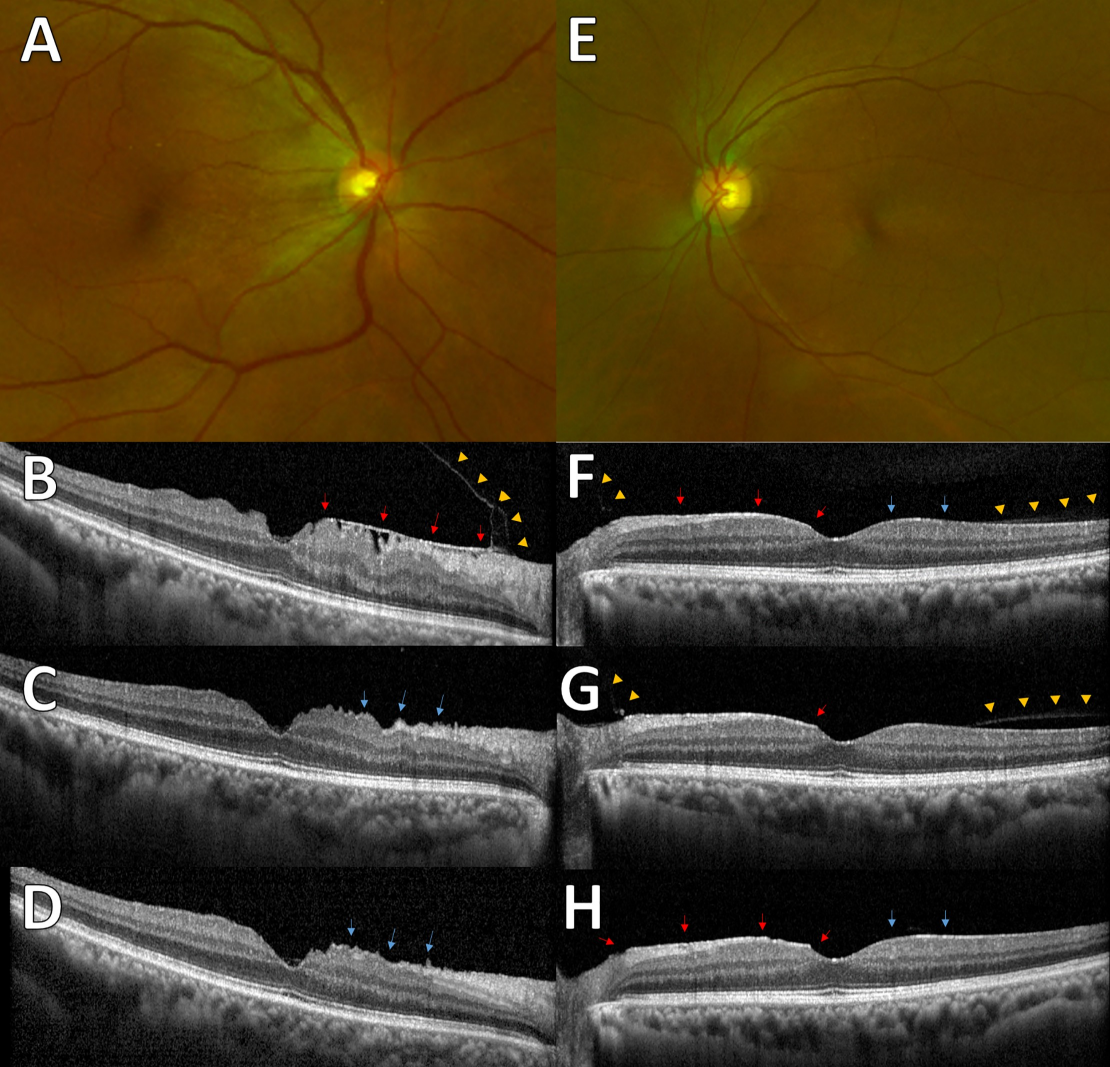
Representative images from subjects with follow-up data during the PVD period. (A) Fundus image shows epiretinal membrane (ERM) at the nasal side. (B) The spectral-domain optical coherence tomography (SD-OCT) image shows ERM at the nasal side (arrows) with attached posterior hyaloid membrane (PHM; arrowheads). (C) After 2 months, the ERM was spontaneously removed with posterior vitreous detachment (PVD). The observed wrinkling of underlying retina (arrows) and (D) 10 months later, the slightly improved wrinkling of retina (arrows). (E) Fundus images show mild ERM. (F) ERM (red arrows) was observed at the nasal retina and epiretinal hyperreflective changes (blue arrows) were observed at the temporal retina. Partial detachment of PHM (arrowheads) was found at the margin of ERM and epiretinal hyperreflective changes. (G) A year later, the detachment of PHM progressed (arrowheads) and retinal peaking at the nasal side of fovea (arrow) was observed. (H) Three months later, PVD was observed and the ERM (red arrows) and hyperreflective changes (blue arrows) remained.

Fig 2 and S2 Table shows the pattern of PVD based on age. The proportion of no PVD and broad VMA decreased and the PVD increased as the patient aged. To evaluate the relationship between VMI and ERM, the proportion of each grade of ERM based on VMI status is shown in Fig 3 and S3 Table. The prevalence and severity of ERM increased as the VMI changed from no PVD to PVD. Broad VMT and focal VMT were not expressed due to the small number of subjects in the categories. Although most eyes with no PVD did not show evidence of ERM, 9 of 737 eyes with no PVD showed various grades of ERM. Fig 4 shows the representative images of eyes having ERM without PVD. ERM started at the focal vitreous detachment and extended along the posterior hyaloid membrane (PHM). Among the 9 eyes with ERM without PVD, 4 eyes had follow-up data including the PVD period. The ERM remained in 2 eyes (50%) after PVD, and in the other 2 eyes (50%), the ERM was removed with PVD (Fig 5).

**Fig 6:**
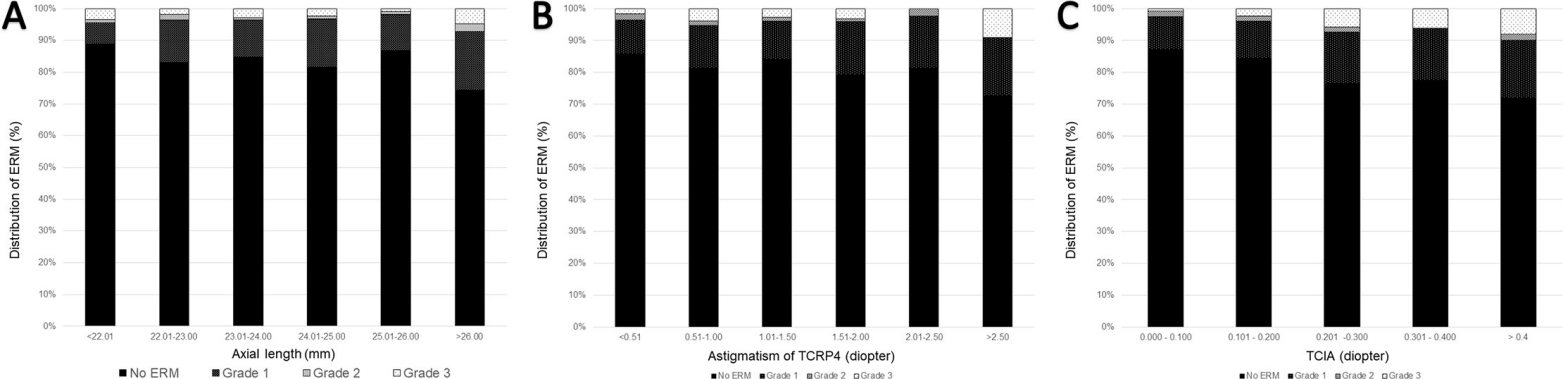
Bar graphs showing the distribution of epiretinal membrane (ERM) based on (A) axial length (AXL), (B) Astigmatism of total corneal refractive power at 4 mm (TCRP4), and (C) total corneal irregular astigmatism (TCIA).

Among the ocular parameters, patients with ERM showed longer AXL and higher corneal astigmatism than the age-matched controls. Fig 6 and S4 Table shows the distribution of ERM based on the AXL, astigmatism of TCRP4, and TCIA. The prevalence of ERM was highest in eyes with AXL longer than 26.0 mm (Fig 6A), corneal astigmatism higher than 2.5 D (Fig 6B), or TCIA higher than 0.4D (Fig 6C). Multiple logistic regression analysis was performed to evaluate whether the AXL, astigmatism of TCRP4, and TCIA were independently associated with the presence of ERM. Univariate logistic regression analysis showed age, PVD, CDVA, astigmatism of TCRP4, TCIA, and LT were associated with the presence of ERM (P < 0.001, P < 0.001, P = 0.001, P = 0.016, P < 0.001, and P = 0.049, respectively). Multiple logistic regression analysis showed age, PVD, CDVA, and TCIA were independently associated with the presence of ERM in phakic eyes (P < 0.001, P < 0.001, P = 0.011, and P = 0.002, respectively; Table 2).

**Table 2:** Univariate analysis and Multiple logistic regression analysis for the presence of ERM in phakic eyes.

	Univariate logistic regression analysis			Multiple logistic regression analysis		
	OR	95% CI	P value	OR	95% CI	P value
Age, years	1.152	1.128–1.176	< 0.001	1.119	1.092–1.147	< 0.001
Gender, male (%)	1.494	0.430–5.192	0.528			
PVD, yes (%)	28.481	14.622–55.476	< 0.001	18.019	9.192–35.323	< 0.001
UDVA, LogMAR	0.916	0.624–1.344	0.654			
CDVA, LogMAR	4.830	1.988–11.738	0.001	3.545	1.336–9.404	0.011
SE, D	0.976	0.941–1.013	0.202			
IOP, mmHg	0.694	0.447–1.076	0.102			
WTW, mm	0.849	0.633–1.139	0.274			
Km of TCRP4, D	0.992	0.925–1.065	0.833			
Astigmatism of TCRP4, D	1.249	1.042–1.496	0.016			
TCIA, D	14.151	3.449–58.058	< 0.001	12.172	2.461–60.204	0.002
Pupil diameter, mm	0.969	0.838–1.202	0.969			
AXL, mm	1.067	0.980–1.163	0.137			
ACD, mm	0.782	0.566–1.080	0.135			
LT, mm	1.420	1.001–2.014	0.049			

ACD = anterior chamber depth; AXL = axial length; CDVA = corrected distance visual acuity; CI = confidential interval; ERM = epiretinal membrane; IOP = intraocular pressure; Km = mean keratometric value; LT = lens thickness; OR = odds ratio; PVD = posterior vitreous detachment; SE = spherical equivalent; TCIA = total corneal irregular astigmatism; TCRP4 = total corneal refractive power at 4 mm; UDVA = uncorrected distance visual acuity; WTW = white to white corneal diameter.

## Discussion

In the present study, the prevalence of ERM was 18.2% of the phakic eyes (mean age 58.72 years). The prevalence of ERM in the current study was lower than in previous population-based studies using SD-OCT. In the Beaver Dam Eye Study (BDES) [7], which was conducted in the USA population (mean age 74.1 years, both phakic and pseudophakic), the prevalence of ERM was 34.1% of the enrolled eyes. The Alienor study [8], which was conducted in the French population (mean age 79.4 years, both phakic and pseudophakic), the prevalence was 69.9% of the enrolled eyes. The higher prevalence of ERM in the Alienor study than in BDES may have resulted from a different ERM classification system. In the present study, the same classification system was used as in the Alienor study to evaluate characteristics of early ERM. We speculated the difference in ERM prevalence between our study and the Alienor study may be due to the younger age and phakic status of the enrolled subjects or the ethnicity.

Information regarding the factors associated with primary ERM is limited. Age, cataract surgery, and PVD are considered directly associated with the development of ERM. In addition to the well-known risk factors, such as age and PVD status, several ocular parameters including astigmatism of TCRP4, TCIA, pupil diameter, AXL, and LT were significantly different between eyes with and without ERM. Based on data after controlling for age and any ocular surgery, eyes with ERM showed higher prevalence of PVD, longer AXL, higher astigmatism of TCRP4, and higher TCIA than the control eyes. Some factors may depend on the PVD status because the PVD develops earlier in myopic eyes with bigger axial length [20]. Multivariate logistic regression analysis showed that CDVA, corneal astigmatism and TCIA were independently correlated with ERM in addition to age and PVD. In multiple logistic regression analysis, AXL did not show statistical significance, indicating the AXL was not a independent factor in PVD. We speculated that the significant correlation of CDVA was because the ERM deteriorated the CDVA in those eyes, rather than the CDVA was an independent factor for the ERM.

Although age and PVD are expected to be associated with ERM, the independent association between TCIA with ERM was unexpected. TCIA is a well-known index for total higher order aberrations (HOAs) that is calculated automatically in Cataract Pre-Op map of Pentacam [21]. HOAs are part of refractive errors that deteriorate retinal images but are not correctable with sphere and cylinder corrections. HOAs were reportedly associated with myopia [22, 23] or ethnicity [24]. Notably, the prevalence of both ERM [25] and HOAs [24] was higher in Asians than in Caucasians. A genetic background regarding the type IV collagen which is exclusively found in the basement membrane of the corneal epithelium/Descemet’s membrane [26], ERM [27], and internal limiting membrane (ILM) [28] may play a role in the higher prevalence of ERM and high corneal astigmatism/HOAs. Further study is warranted to elucidate the mechanism.

The general consensus is that ERM develops after PVD, however, there were nine eyes showing ERM without PVD in the present study. Among those nine eyes, four eyes had follow-up data during the period of PVD. Notably, half of the cases showed spontaneous resolution and half of the cases showed ERM remained after PVD. The spontaneous resolution of ERM has been reported in the literature [29]. Potentially, both PHM and ILM could be the niche for the cellular proliferation. We speculated that the ERM could be removed if the cellular proliferation was based on the PHM, and would remain if the cellular proliferation was based on the ILM. A clinicopathologic study is warranted to confirm the hypothesis.

The present study was limited because this was a clinic-based study and the cohort may not represent the entire population. Further study is warranted to understand the cause and aggravating factors of primary ERM.

## Supporting information

S1 Table Clinical characteristics and ocular examination data of all population. (DOCX)

S2 Table Distribution of vitreomacular interface according to age. (DOCX)

S3 Table Distribution of epiretinal membrane according to vitreomacular interface. (DOCX)

S4 Table Distribution of epiretinal membrane according to ocular parameters. (DOCX)
