# Supplementary material for: Prevalence of epiretinal membrane in the phakic eyes based on spectral-domain optical coherence tomography
Source: PLoS One. 2021 Jan 7;16(1):e0245063. doi: 10.1371/journal.pone.0245063 (PMC7790294; doi:10.1371/journal.pone.0245063)
Supplement: S1 Table — (DOCX) [file pone.0245063.s001.docx]

S1 Table. Clinical characteristics and ocular examination data of all population.

|  | All population (n=2354) |
| --- | --- |
| Age, years | 58.72 ± 5.73 |
| Gender, male (%) | 1180 (50.1) |
| ERM, yes (%) | 429 (18.2) |
| Stage 1 | 304 (12.9) |
| Stage 2 | 37 (1.6) |
| Stage 3 | 90 (3.8) |
| PVD, yes (%) | 1617 (68.7) |
| Focal VMA | 270 (11.5) |
| Broad VMA | 573 (24.3) |
| Focal VMT | 6 (0.2) |
| Broad VMT | 1 (0.0) |
| PVD | 767 (32.6) |
| UDVA, LogMAR | 0.34 ± 0.30 |
| CDVA, LogMAR | 0.03 ± 0.10 |
| SE, D | -0.15 ± 2.74 |
| IOP, mmHg | 15.43 ± 2.68 |
| WTW, mm | 11.40 ± 0.38 |
| Km of TCRP4, D | 43.64 ± 1.53 |
| Astigmatism of TCRP4, D | 0.79 ± 0.56 |
| TCIA, D | 0.16 ± 0.07 |
| Pupil diameter, mm | 3.69 ± 0.72 |
| AXL, mm | 23.82 ± 1.27 |
| ACD, mm | 3.16 ± 0.35 |
| LT, mm | 4.44 ± 0.32 |

ACD = anterior chamber depth; AXL = axial length; CDVA = corrected distance visual acuity; Km = mean keratometric value; LT = lens thickness; PVD = posterior vitreous detachment; SE = spherical equivalent; TCIA = total corneal irregular astigmatism; TCRP = total corneal refractive power; WTW = white to white. Data are mean ± standard deviation unless otherwise indicated.
