# Supplementary material for: Prevalence of epiretinal membrane in the phakic eyes based on spectral-domain optical coherence tomography
Source: PLoS One. 2021 Jan 7;16(1):e0245063. doi: 10.1371/journal.pone.0245063 (PMC7790294; doi:10.1371/journal.pone.0245063)
Supplement: S2 Table — (DOCX) [file pone.0245063.s002.docx]

S2 Table. Distribution of vitreomacular interface according to age (N=2354).

| VMI | Age (years) | | | | | |
| --- | --- | --- | --- | --- | --- | --- |
|  | < 51 (N=166) | 51 - 55 (N=567) | 56 - 60 (N=705) | 61 - 65 (N=609) | 66 - 70 (N=260) | > 70 (N=47) |
| No PVD | 87 (52.4) | 253 (44.6) | 239 (33.9) | 122 (20) | 36 (13.8) | 0 (0.0) |
| Focal VMA | 6 (3.6) | 42 (7.4) | 80 (11.3) | 110 (18.1) | 31 (11.9) | 1 (2.1) |
| Diffuse VMA | 62 (37.3) | 182 (32.1) | 201 (28.5) | 99 (16.3) | 26 (10.0) | 3 (6.4) |
| Focal VMT | 0 (0.0) | 1 (0.2) | 0 (0.0) | 2 (0.3) | 1 (0.4) | 2 (4.3) |
| Diffuse VMT | 0 (0.0) | 1 (0.2) | 0 (0.0) | 0 (0.0) | 0 (0.0) | 0 (0.0) |
| PVD | 11 (6.6) | 88 (15.5) | 185 (26.2) | 276 (45.3) | 166 (63.8) | 41 (87.2) |

PVD = posterior vitreous detachment; VMA = vitreomacular attachment; VMI = vitreomacular interface; VMT = vitreomacular traction

Data are number (%) unless otherwise indicated.
