# Supplementary material for: Prevalence of epiretinal membrane in the phakic eyes based on spectral-domain optical coherence tomography
Source: PLoS One. 2021 Jan 7;16(1):e0245063. doi: 10.1371/journal.pone.0245063 (PMC7790294; doi:10.1371/journal.pone.0245063)
Supplement: S3 Table — (DOCX) [file pone.0245063.s003.docx]

S3 Table. Distribution of epiretinal membrane according to vitreomacular interface (N=2354).

| VMI | Grade of ERM | | | |
| --- | --- | --- | --- | --- |
|  | No ERM | Grade 1 | Grade 2 | Grade 3 |
| No PVD (N = 736) | 727 (98.8) | 7 (0.9) | 1 (0.1) | 1 (0.1) |
| Diffuse VMA (N = 573) | 534 (93.2) | 26 (4.5) | 4 (0.7) | 9 (1.6) |
| Focal VMA (N = 270) | 221 (81.9) | 40 (14.8) | 1 (0.4) | 8 (3.0) |
| Diffue VMT (N = 2) | 1 (50.0) | 0 (0.0) | 0 (0.0) | 1 (50.0) |
| Focal VMT (N = 6) | 4 (66.7) | 2 (33.3) | 0 (0.0) | 0 (0.0) |
| PVD (N = 767) | 440 (57.4) | 225 (29.3) | 31 (4.0) | 71 (9.3) |

ERM = epiretinal membrane; PVD = posterior vitreous detachment; VMA = vitreomacular attachment; VMI = vitreomacular interface; VMT = vitreomacular traction. Data are number (%) unless otherwise indicated.
