# Supplementary material for: Prevalence of epiretinal membrane in the phakic eyes based on spectral-domain optical coherence tomography
Source: PLoS One. 2021 Jan 7;16(1):e0245063. doi: 10.1371/journal.pone.0245063 (PMC7790294; doi:10.1371/journal.pone.0245063)
Supplement: S4 Table — (DOCX) [file pone.0245063.s004.docx]

S4 Table. Distribution of epiretinal membrane according to ocular parameters (N=2354).

| Variables | Grade of ERM | | | |
| --- | --- | --- | --- | --- |
|  | No ERM | Grade 1 | Grade 2 | Grade 3 |
| Axial length (mm) |  |  |  |  |
| < 22.01 (N=91) | 81 (89.0) | 6 (6.6) | 1 (1.1) | 3 (3.3) |
| 22.01-23.00 (N=531) | 439 (82.6) | 74 (13.9) | 9 (1.8) | 9 (1.8) |
| 23.01-24.00 (N=852) | 723 (84.9) | 100 (11.7) | 7 (0.8) | 22 (2.6) |
| 24.01-25.00 (N=521) | 436 (83.6) | 70 (13.4) | 4 (0.8) | 11 (2.2) |
| 25.01-26.00 (N=220) | 190 (86.4) | 26 (11.8) | 2 (0.9) | 2 (0.9) |
| > 26.00 (N=139) | 98 (74.8) | 25 (19.1) | 2 (1.5) | 6 (4.6) |
| Astigmatism of TCRP4 (D) |  |  |  |  |
| < 0.51 (N=917) | 784 (85.5) | 100 (10.9) | 17 (1.9) | 16 (1.7) |
| 0.51-1.00 (N=878) | 710 (80.8) | 121 (13.8) | 12 (1.4) | 35 (4.0) |
| 1.01-1.50 (N=355) | 299 (84.0) | 44 (12.5) | 4 (1.2) | 8 (2.3) |
| 1.51-2.00 (N=127) | 100 (78.7) | 22 (17.3) | 1 (0.8) | 4 (3.1) |
| 2.01-2.50 (N=44) | 36 (81.8) | 7 (15.9) | 1 (2.3) | 0 (0.0) |
| > 2.50 (N=33) | 24 (72.7) | 6 (18.2) | 0 (0.0) | 3 (9.1) |
| TCIA (D) |  |  |  |  |
| 0.000-0.100 (N=462) | 401 (86.8) | 49 (10.6) | 9 (1.9) | 3 (0.6) |
| 0.101-0.200 (N=1469) | 1235 (84.1) | 172 (11.7) | 22 (1.5) | 40 (2.7) |
| 0.201-0.300 (N=348) | 263 (75.6) | 60 (17.2) | 4 (1.1) | 21 (6.0) |
| 0.301-0.400 (N=50) | 32 (64.0) | 14 (28.0) | 1 (2.0) | 3 (6.0) |
| > 0.400 (N=25) | 19 (76.0) | 6 (24.0) | 0 (0.0) | 0 (0.0) |

D = diopter; ERM = epiretinal membrane; TCRP4 = total corneal refractive power at 4 mm; TCIA = total corneal irregular astigmatism. Data are number (%) unless otherwise indicated.
